
JOURNAL INFORMATION
==============================
NLM Title Abbreviation: Access Microbiol
Journal ID: Access Microbiol
NLM Journal ID: 101746927
Journal ID: acmi

Access Microbiology

EISSN: 2516-8290
Publisher: Microbiology Society

ARTICLE INFORMATION
==============================
PMCID: PMC7481740
PMID: 32974537
Article version: 1
Subjects: Oral Abstract

Abstracts from the Anaerobe Focused Meeting 2019

Publication date: 2019
Electronic publication date: 2019 Sep 30
Volume: 1
Issue: 7
Electronic Location ID: 11
Copyright: © 2019 The Authors
Copyright year: 2019
License: This is an open-access article distributed under the terms of the Creative Commons Attribution License.
License URL: https://creativecommons.org/licenses/by/4.0/

==============================


The anaerobic skin colonizer Staphylococcus saccharolyticus, a possible agent of prosthetic joint infections, exhibits excessive genome decay

http://jmmcr.microbiologyresearch.org

Brüggemann Holger 1 *
Söderquist Bo 2
Aarhus University , Aarhus , Denmark
Örebro University , Örebro , Sweden
*Correspondence:Holger Brüggemann, brueggemann@biomed.au.dk

The use of MALDI-TOF MS as a rapid test to detect carbapenem resistance in Bacteroides fragilis

http://jmmcr.microbiologyresearch.org

Copsey-Mawer Sarah *
Scotford Selina
Copsey-Mawer Bethan
Davis Carol
Perry Michael
Morris Trefor
Hughes Harriet
UK Anaerobe Reference Unit , Public Health Wales , Cardiff
*Correspondence:Sarah Copsey-Mawer, sarahcopsey@hotmail.com

